# Supplementary material for: Cancer-Associated Fibroblasts Promote Tumor Aggressiveness in Head and Neck Cancer through Chemokine Ligand 11 and C-C Motif Chemokine Receptor 3 Signaling Circuit
Source: Cancers (Basel). 2022 Jun 27;14(13):3141. doi: 10.3390/cancers14133141 (PMC9264987; doi:10.3390/cancers14133141)
Supplement: Supplementary file 1 [file cancers-14-03141-s001.zip › supplementary materials.pdf]

Supporting information

Supplementary material S1

Analyzing western blots with ImageJ

Band intensity was also quantified by ImageJ software. The films were obtained images were converted to 8-bit format to perform uncalibrated optical density (OD). After conversion, the background was subtracted through the rolling ball visualized (with the option “create background don't subtract” activated). In this manner, it is possible to determine the background of each image even when background is not uniform. Finally, the obtained image (negative) was inverted to generate an image with detect bands and a uniformly subtracted background.

Western blot ratio:

1(b)

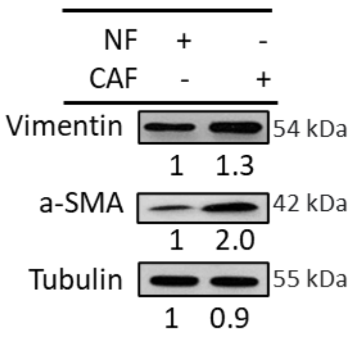

1(f)

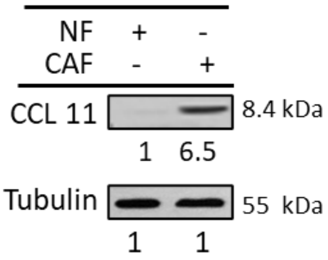

1(g)

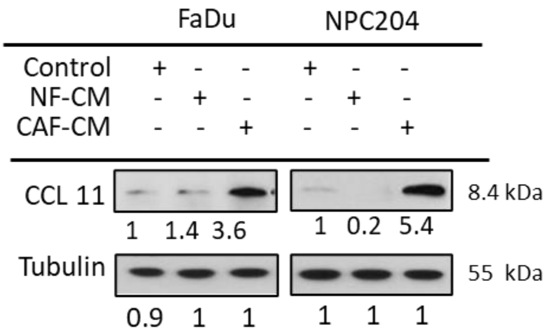
FaDu
NPC204
Control + - - + - -
NF-CM - + - - + -
CAF-CM - - + - - +
CCL 11
8.4 kDa
1 1.4 3.6
1 0.2 5.4
Tubulin
55 kDa
0.9 1 1
1 1 1

2(C)

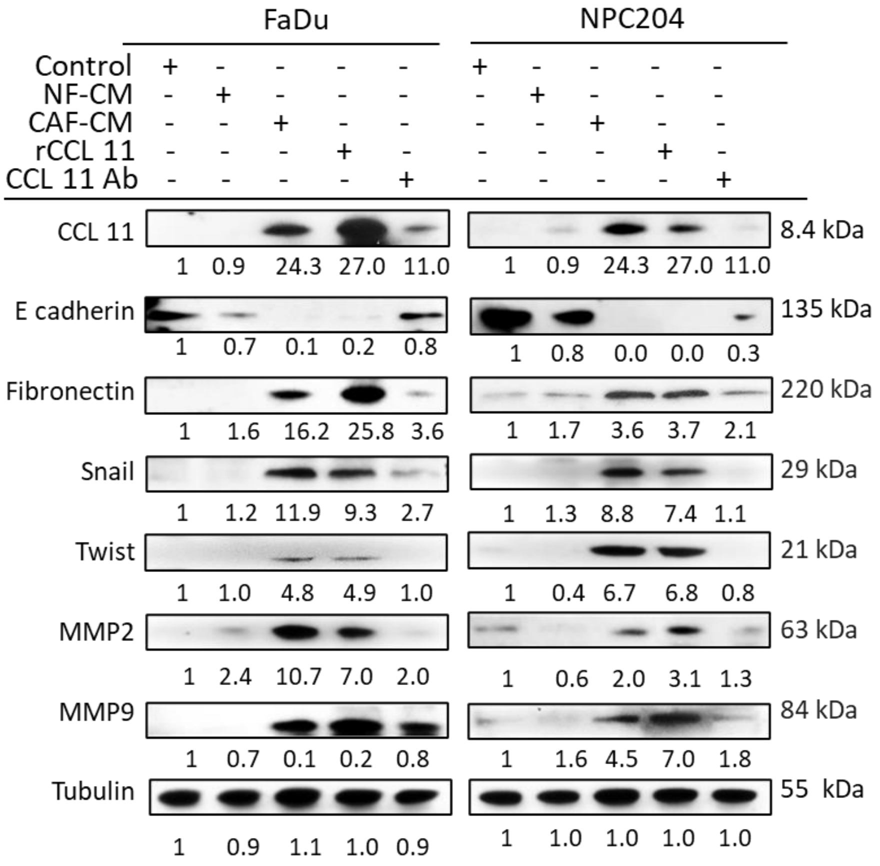
FaDu
NPC204
Control + - - - - + - - - -
NF-CM - + - - - - + - - -
CAF-CM - - + - - - - + - -
rCCL 11 - - - + - - - - + -
CCL 11 Ab - - - - + - - - - +
CCL 11
1 0.9 24.3 27.0 11.0
1 0.9 24.3 27.0 11.0
8.4 kDa
E cadherin
1 0.7 0.1 0.2 0.8
1 0.8 0.0 0.0 0.3
135 kDa
Fibronectin
1 1.6 16.2 25.8 3.6
1 1.7 3.6 3.7 2.1
220 kDa
Snail
1 1.2 11.9 9.3 2.7
1 1.3 8.8 7.4 1.1
29 kDa
Twist
1 1.0 4.8 4.9 1.0
1 0.4 6.7 6.8 0.8
21 kDa
MMP2
1 2.4 10.7 7.0 2.0
1 0.6 2.0 3.1 1.3
63 kDa
MMP9
1 0.7 0.1 0.2 0.8
1 1.6 4.5 7.0 1.8
84 kDa
Tubulin
1 0.9 1.1 1.0 0.9
1 1.0 1.0 1.0 1.0
55 kDa

3(d)

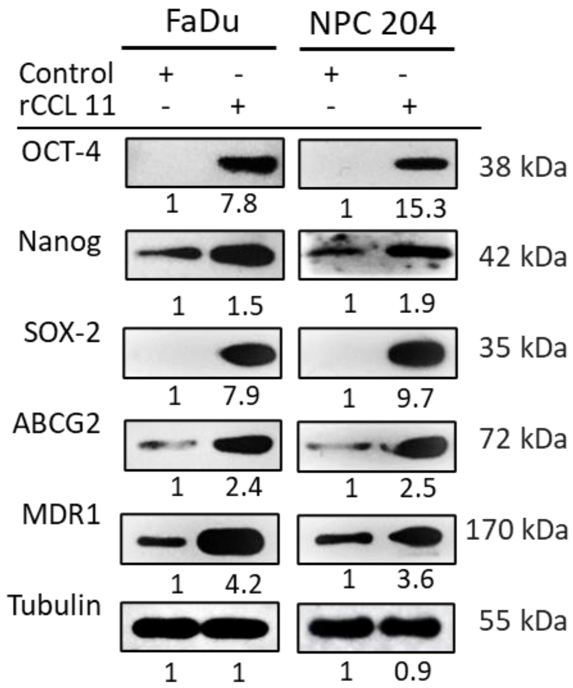
FaDu
NPC 204
Control + - + -
rCCL 11 - + - +
OCT-4
1 7.8
1 15.3
38 kDa
Nanog
1 1.5
1 1.9
42 kDa
SOX-2
1 7.9
1 9.7
35 kDa
ABCG2
1 2.4
1 2.5
72 kDa
MDR1
1 4.2
1 3.6
170 kDa
Tubulin
1 1
1 0.9
55 kDa

4(b)

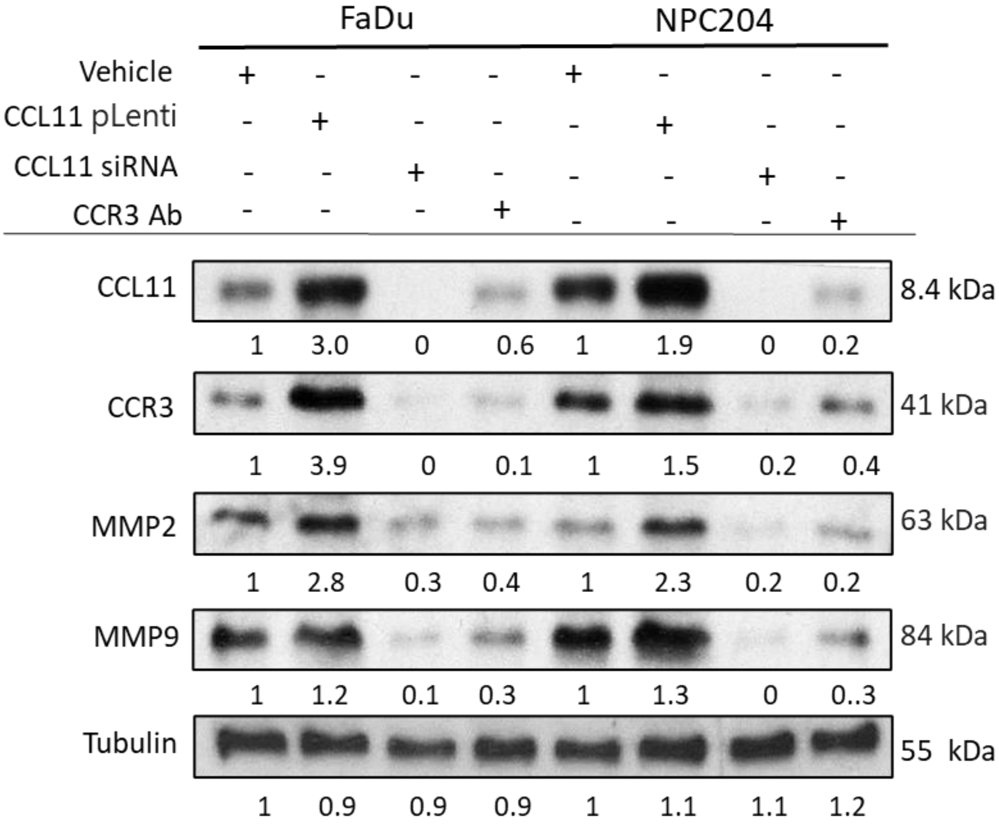
FaDu
NPC204
Vehicle + - - - + - - -
CCL11 pLenti - + - - - + - -
CCL11 siRNA - - + - - - + -
CCR3 Ab - - - + - - - +
CCL11
1 3.0 0 0.6 1 1.9 0 0.2
8.4 kDa
CCR3
1 3.9 0 0.1 1 1.5 0.2 0.4
41 kDa
MMP2
1 2.8 0.3 0.4 1 2.3 0.2 0.2
63 kDa
MMP9
1 1.2 0.1 0.3 1 1.3 0 0..3
84 kDa
Tubulin
1 0.9 0.9 0.9 1 1.1 1.1 1.2
55 kDa

Supplementary material S2

A generic plasmid map for the pLenti-GIII-CMV-Luc-2A-Puro lentiviral expression vector backbone and LVP110251 (CCL11 sequence): Coding sequence: 291bp

ATGAAGGTCTCCGCAGCACTTCTGTGGCTGCTGCTCATAGCAGCTGCCTTC
AGCCCCCAGGGGCTCGCTGGGCCAGCTTCTGTCCCAACCACCTGCTGCTTT
AACCTGGCCAATAGGAAGATACCCCTTCAGCGACTAGAGAGCTACAGGAG
AATCACCAGTGGCAAATGTCCCCAGAAAGCTGTGATCTTCAAGACCAAAC
TGGCCAAGGATATCTGTGCCGACCCCAAGAAGAAGTGGGTGCAGGATTCC
ATGAAGTATCTGGACCAAAAATCTCCAACTCCAAAGCCA

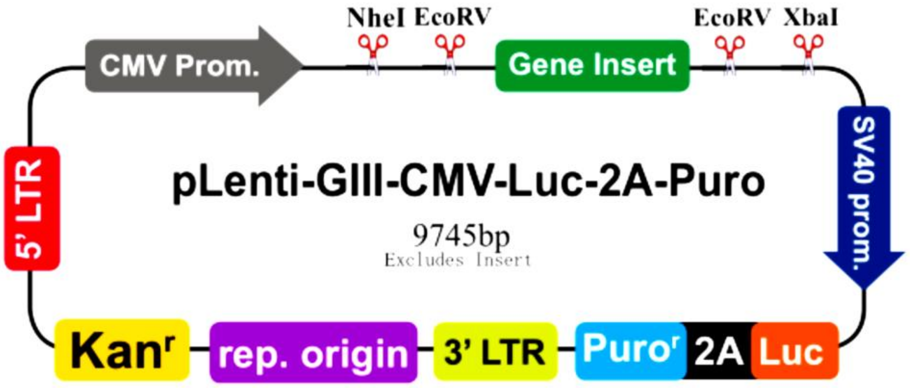

Supplementary Tables

Table S1. List of antibodies used in this study

**Protein**	Dilution	**Assay**	**Origin**
rCCL11			MBS142051, MYBioSource
CCR3	1:1000	WB/IHC	GTX100342; GeneTex
CCL11	1:1000/1:2000/1:100	WB/ELISA/HC	MAB320; R&D System
α-Smooth muscle actin	1:200	WB	ab5694; abcam
Vimentin	1:300	WB	GTX45117; Gene Tex
MMP2	1:200	WB	MAB3308, Merck
MMP9	1:200	WB	bs-0397R, Bioss Antibodies
p38MAPK	1:200	WB	GTX110720, GeneTex
pp38MAPK	1:200	WB	GTX133460, GeneTex
ERK	1:200	WB	bs-0022R, Bioss Antibodies
pERK	1:200	WB	bs-1522R, Bioss Antibodies
JNK	1:200	WB	bs-2592R, Bioss Antibodies
pJNK	1:200	WB	bs-1640R, Bioss Antibodies
Snail	1:100	WB	GTX100754; Gene Tex
Twist	1:100	WB	GTX127310; Gene Tex
SOX2	1:100	WB	GTX101507; Gene Tex
OCT 4	1:100	WB	GTX627419; Gene Tex
Nanog	1:100	WB	GTX100863; Gene Tex
ABCG2	1:200	WB	Sc-69988; Santa Cruz Biotechnology
MDR1	1:200	WB	Sc-55510; Santa Cruz Biotechnology
Ecadherin	1:200	WB	Cat #13-5700; Thermo Fisher Scientific Inc.
Fibronectin	1:200	WB	Sc-271098; Santa Cruz Biotechnology
CD10	1:1000	Flow	MAB10254, R&D systems, Minneapolis, MN, USA
GPR77	1:1000	Flow	GTX78263, Genetex, California,USA
CD24	1:200	Flow	IM1428U, Beckman Coulter, Inc
CD44	1:100	Flow	A32537, Beckman Coulter, Inc
AlexaFluor488	1:1000	Flow	ab150113; abcam
AlexaFluor647	1:1000	Flow	ab150075; abcam
β-Tubulin	1:2000	WB	Sc-5274; Santa Cruz Biotechnology

Table S2. List of primers used in this study

Name	Sequence
Vimentin	F: 5′- AATGACCGCTTCGCCAACT-3'<br>R: 5′- ATCTTATTCTGCTGCTCCAGGAA-3'
αSMA	F: 5′-CTATGCCTCTGGACGCACAACT-3'<br>R: 5′-CAGATCCAGACGCATGATGGCA-3'
CCL11	F: 5′- GCTACAGGAGAATCACCAGTGG-3'<br>R: 5′- GGAATCCTGCACCCACTTCTTC-3'
GAPDH	F: 5′-TCCACTGGCGTCTTCACC-3'<br>R: 5′-GGCAGAGATGATGACCCTTTT-3'

Table S3. Patient characteristics in survival analysis of CCL11 and CCR3 expression

	All patients, no (%)	CCL11, no (%)			CCR3, no (%)			Double positive, no (%)		
		negative	positive	p-value	negative	positive	p-value	negative	positive	p-value
No. of patients	104 (100)	49(47.1)	55(52.9)		76(73.1)	28(26.9)		78(75.0)	26(25.0)	
Age, mean ± SD, years	54.6 ± 11.8			0.297			0.039*			0.023*
≥50	69(66.3)	30(28.8)	39(37.5)		46(44.2)	23(22.1)		47(45.2)	22(21.2)	
<50	35(33.7)	19(18.3)	16(15.4)		30(28.8)	5(4.8)		31(29.8)	4(3.8)	
Sex				0.254			0.327			0.249
male	94(90.4)	46(44.2)	48(46.2)		70(67.3)	24(23.1)		72(69.2)	22(21.2)	
female	10(9.6)	3(2.9)	7(6.7)		6(5.8)	4(3.8)		6(5.8)	4(3.8)	
Smoking				0.919			0.511			0.691
yes	79(76.0)	37(35.6)	42(40.4)		59(56.7)	20(19.2)		60(57.7)	19(18.3)	
no	25(24.0)	12(11.5)	13(12.5)		17(16.3)	8(7.7)		18(17.3)	7(6.7)	
Alcohol, yes				0.215			0.054			0.053
yes	57(54.8)	30(28.8)	27(26.0)		46(44.2)	11(10.6)		47(45.2)	10(9.6)	
no	47(45.2)	19(18.3)	28(26.9)		30(28.8)	17(16.3)		31(29.8)	16(15.4)	
Betel nut, yes				0.059			0.247			0.085
yes	43(41.3)	25(24.0)	18(17.3)		34(32.7)	9(8.7)		36(34.6)	7 (6.7)	
no	61(58.7)	24(23.1)	37(35.6)		42(40.4)	19(18.3)		42(40.4)	19(18.3)	
Primary sites				0.833			0.730			0.566
Oral cavity	84 (80.8)	40(38.5)	44(42.3)		62(59.6)	22(21.2)		64(61.5)	20(19.2)	
Others	20 (19.2)	9(8.7)	11(10.6)		14(13.5)	6(5.8)		14(13.5)	6(5.8)	
T stage				0.060			0.085			0.022*
1-2	60(57.7)	33(31.7)	27(26.0)		45(43.3)	15(14.4)		50(48.1)	10(9.6)	
3-4	44(42.3)	16(15.4)	28(26.9)		26(25.0)	18(17.3)		28(26.9)	16(15.4)	
N stage				0.301			0.257			0.719
0	69(66.3)	35(33.7)	34(32.7)		48(46.2)	28(26.9)		51(49.0)	18(17.3)	
1-3	35(33.7)	14(13.5)	21(20.2)		21(20.2)	7(6.7)		27(26.0)	8(7.7)	
M stage				NA			NA			NA
0	104(100)	49(47.1)	55(52.9)		76(73.1)	28(26.9)		78(75.0)	26(25.0)	

1	0	0(0)	0(0)		0(0)	0(0)		0(0)	0(0)	
Stage				0.034*			0.973			0.173
I/II	48(46.2)	28(26.9)	20(19.2)		35(33.7)	13(12.5)		39(37.5)	9(8.7)	
III/IV	56(53.8)	21(20.2)	35(33.7)		41(39.4)	15(14.4)		39(37.5)	17(16.3)	
Treatment				0.147			0.423			0.105
Surgery	45 (43.3)	36(25.0)	19(18.3)		35(33.7)	10(9.6)		38(36.5)	7(6.7)	
(CC)RT	4 (3.8)	2(1.9)	2(1.9)		2(1.9)	2(1.9)		2(1.9)	2(1.9)	
Surgery + (CC)RT	55 (52.9)	21(20.2)	34(32.7)		39(37.5)	16(15.4)		38(36.5)	17(16.3)	
Surgical margin				0.753			0.590			0.158
≥3mm	34(64.4)	34(32.7)	33(31.7)		48(46.2)	19(18.3)		52(50.0)	15(14.4)	
<3mm	26(25.0)	10(9.6)	16(15.4)		20(19.2)	6(5.8)		17(16.3)	9(8.7)	
No surgery or missing	11(10.5)	5(4.8)	6(5.79)		8(7.7)	3(2.9)()		9(8.6)	2(1.9)	
ECE				0.103			0.595			0.478
yes	12(11.5)	3(2.9)	9(8.7)		8(7.7)	4(3.8)		8(7.7)	4(3.8)	
no	92(88.5)	46(44.2)	16(44.2)		68(65.4)	24(23.1)		70(67.3)	22(21.2)	

Abbreviations: SD = standard deviation, RT = radiotherapy, CCRT = concurrent chemoradiotherapy, ECE = Extracapsular extension

*p-value < 0.05

Table S4. Prognostic factors on overall survival by Cox proportional-hazards model

	Univariable		Multivariable (model 1)†		Multivariable (model 2)†	
	HR (95% CI)	p-value	HR (95% CI)	p-value	HR (95% CI)	p-value
Female vs. male	0.63(0.22-1.81)	0.388				
Age ≥50 years	1.10(0.50-2.41)	0.818				
Smoking, no vs. yes	0.46(0.16-1.33)	0.151				
Alcohol, no vs. yes	0.99(0.48-2.06)	0.980				
Betel nut, no vs. yes	0.76(0.37-1.58)	0.460				
Primary site, oral cavity vs others	1.18(0.48-2.89)	0.721				
T stage, T3,4 vs. T1,2	3.10(1.44-6.69)	0.004*	2.44(1.11-5.38)	0.027*	2.38(1.07-5.27)	0.033*
N stage, N1-3 vs. N0	1.88(0.90-3.94)	0.093	1.61 (0.74-3.52)	0.229	1.77(0.82-3.82)	0.146
Treatment, surgery vs. surgery + (CC)RT	1.16(0.94-1.43)	0.162				
Surgical margin, ≥3mm vs. <3mm	1.37(0.59-3.22)	0.466				
ECE, yes vs.no	1.18(0.41-3.41)	0.767				
CCR3 overexpression, yes vs. no	2.17(1.00-4.70)	0.049*	1.63(0.70-3.80)	0.257		
CCL11 overexpression, yes vs. no	3.41(1.45-8.00)	0.005*	2.61 (1.06-6.42)	0.037*		
CCR3 & CCL11 co-overexpression , yes vs. no	3.31(0.15-7.19)	0.002*			3.15(1.41-7.04)	0.005*

Abbreviations: RT = radiotherapy, CCRT = concurrent chemoradiotherapy, ECE = Extracapsular extension

*p-value < 0.05

† Individual CCL11 or CCR3 expression correlated highly with CCR3 & CCL11 co-expression. Two Cox models were used to avoid collinearity.
