# Supplementary figures and images for: Cancer-Associated Fibroblasts Promote Tumor Aggressiveness in Head and Neck Cancer through Chemokine Ligand 11 and C-C Motif Chemokine Receptor 3 Signaling Circuit
Source: Cancers (Basel). 2022 Jun 27;14(13):3141. doi: 10.3390/cancers14133141 (PMC9264987; doi:10.3390/cancers14133141)

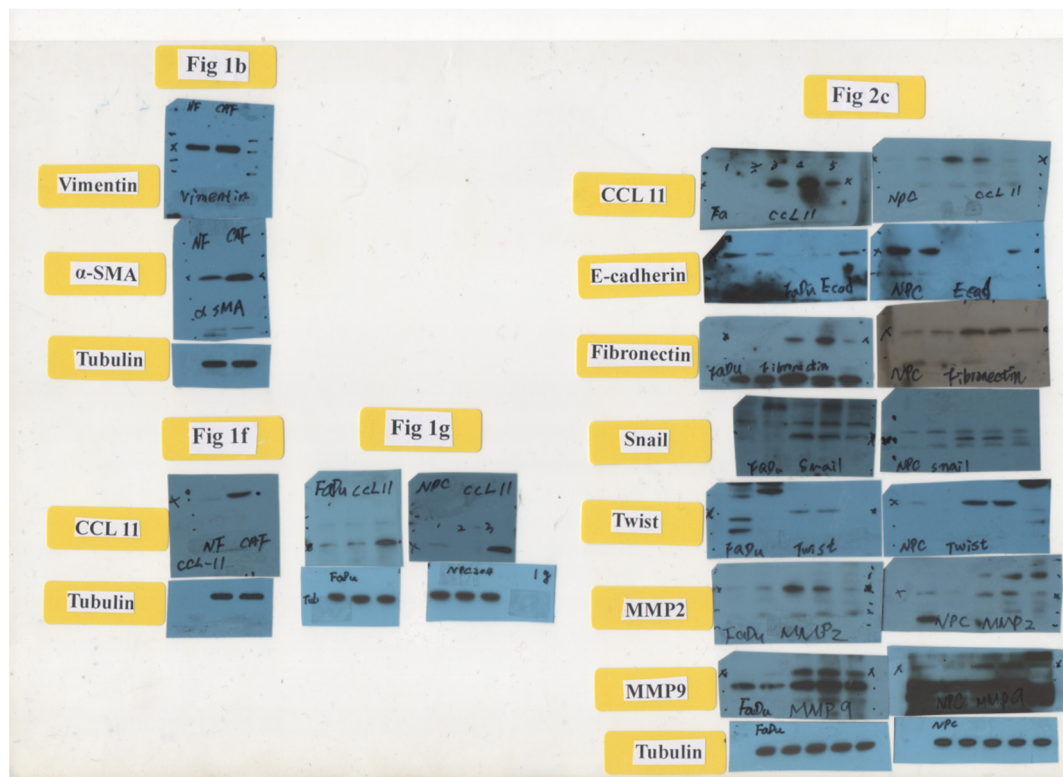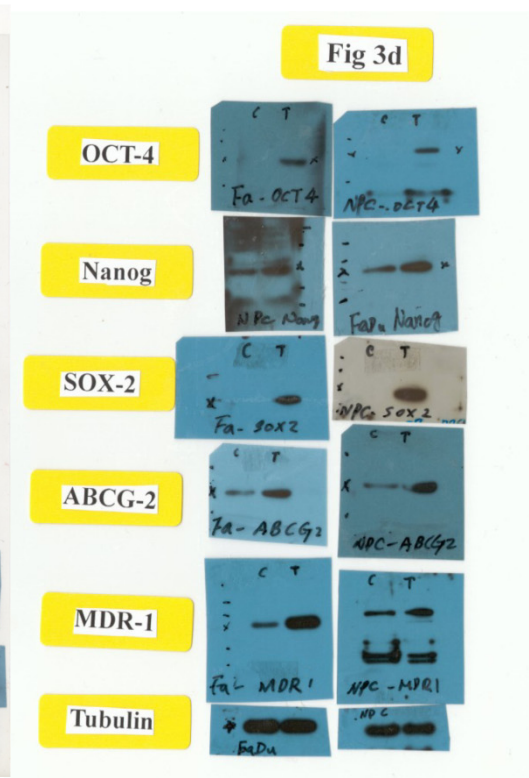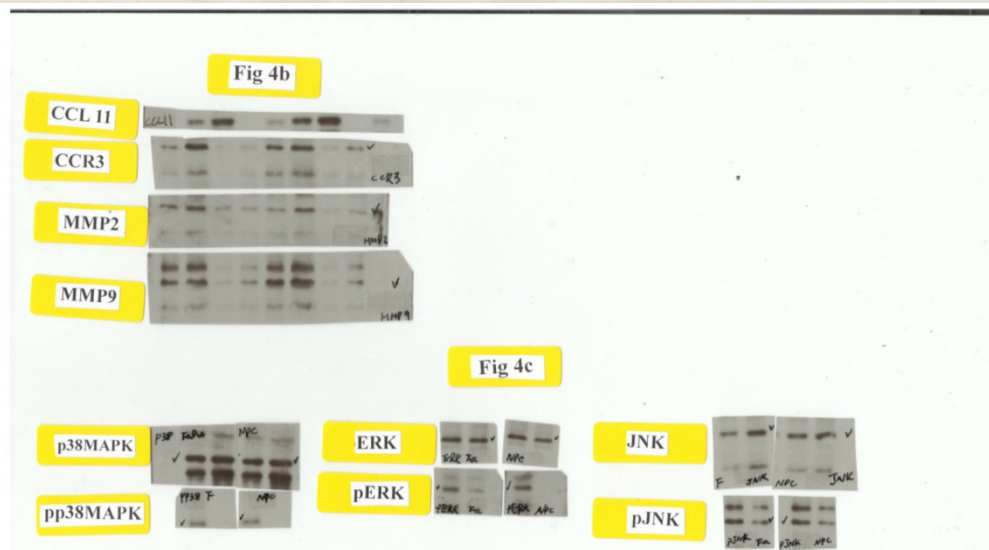

Supplement: Supplementary file 1 [file cancers-14-03141-s001.zip › Figure S1.pdf]

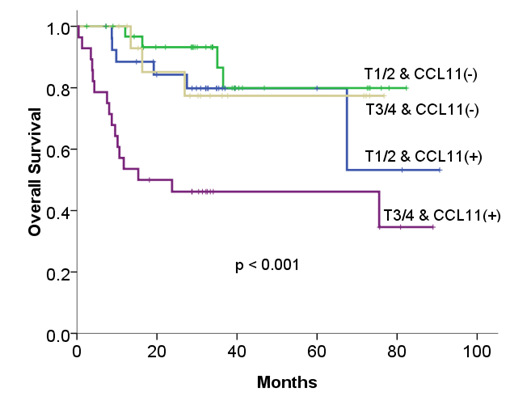

Supplement: Supplementary file 1 [file cancers-14-03141-s001.zip › Figure S2.png]
